# Modulatory role of *Spirulina platensis* in oxidative stress, apoptosis, and gene expression in a rat model of dexamethasone-induced hepatotoxicity

**DOI:** 10.3389/fphar.2025.1610793

**Published:** 2025-08-18

**Authors:** Amina Zedan, Amira M. El-Moslemany, Rasha M. Bahnasy, Hanan Salah Eldeen Eldamaty, Suzan S. Ibraheim, Badriyah S. Alotaibi, Mohamed Abdelmegeid, Mustafa Shukry, Ahmed A. Elolimy

**Affiliations:** ^1^ Department of Biological and Environmental Sciences, Faculty of Home Economics, Al-Azhar University, Tanta, Egypt; ^2^ Nutrition and Food Science Department, Faculty of Home Economics, Al-Azhar University, Tanta, Egypt; ^3^ Department of Pharmaceutical Sciences, College of Pharmacy, Princess Nourah Bint Abdulrahman University, Riyadh, Saudi Arabia; ^4^ Department of Animal Medicine, Faculty of Veterinary Medicine, Kafrelsheikh University, Kafrelsheikh, Egypt; ^5^ Veterinary Program, Faculty of Health Sciences, Higher Colleges of Technology, Sharjah Men’s Campus, Al-Ain, United Arab Emirates; ^6^ Physiology Department, Faculty of Veterinary Medicine, Kafrelsheikh University, Kafrelsheikh, Egypt; ^7^ Department of Integrative Agriculture, College of Agriculture and Veterinary Medicine, United Arab Emirates University, Al Ain, United Arab Emirates

**Keywords:** hyperlipidemia, dexamethasone, *Spirulina platensis*, Nrf2 pathway, PPAR-α, apoptosis

## Abstract

The rising prevalence of hyperlipidemia and hepatic disorders has intensified interest in the therapeutic use of functional foods and botanical drugs. *Spirulina platensis*, a blue-green microalga, is known for its antioxidant, anti-inflammatory, and lipid-lowering properties. However, its potential hepatoprotective effects, particularly against glucocorticoid-induced liver damage, remain underexplored. This study aimed to investigate the protective effects of *Spirulina platensis* aqueous extract (SPAE) against dexamethasone (DEX)-induced oxidative stress, lipid dysregulation, apoptosis, hepatic injury, and associated gene expression changes in male rats. Forty male albino rats (150 ± 10 g) were randomly divided into four groups (*n* = 10). The control group received a standard diet and saline for 28 days. The second group was intraperitoneally injected with DEX (10 mg/kg) on alternate days for 28 days to induce hepatic and oxidative damage. The third and fourth groups were co-administered DEX with SPAE at 400 mg/kg and 800 mg/kg body weight/day orally for the same period. At the end of the experiment, key physiological and biochemical parameters were assessed, including feed intake, body weight gain, feed efficiency ratio (FER), and liver weight. Blood lipid profiles, liver enzymes (ALT, AST, ALP), total and direct bilirubin, and serum protein levels were analyzed. Additionally, antioxidant enzyme activities (SOD, CAT), markers of lipid peroxidation (MDA, NO), and mRNA expression levels of genes related to oxidative stress (Nrf2, SOD2), apoptosis (Bax, Bcl-2), lipid metabolism (PPAR-α), and DNA damage (p53) were evaluated using quantitative RT-PCR. SPAE treatment also modulated upstream regulators Keap1 and AMPK, supporting activation of the Nrf2 and PPAR-α pathways. The results revealed that SPAE significantly ameliorated DEX-induced hyperlipidemia, hepatic dysfunction, oxidative stress, and abnormal gene expression profiles, with the 800 mg/kg dose showing superior efficacy. These findings suggest that *Spirulina platensis* aqueous extract offers a promising protective effect against glucocorticoid-induced metabolic and hepatic disturbances, potentially through its antioxidant, anti-apoptotic, and gene-regulatory properties.

## Graphical Abstract

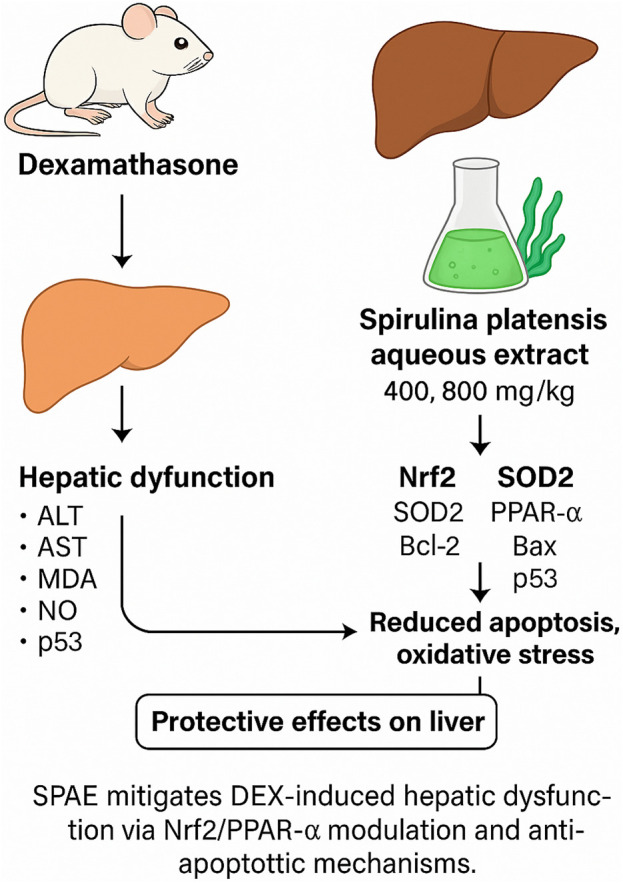

## 1 Introduction

Dexamethasone (DEX) is a potent synthetic glucocorticoid widely used for its anti-inflammatory and immunosuppressive properties. It is frequently prescribed in the management of various medical conditions such as asthma, severe allergies, autoimmune disorders, and various malignancies (Madamsetty et al., 2022; Caramori, 2019). Glucocorticoids, including dexamethasone, are also integral in treating inflammation, inflammatory bowel diseases, allergic reactions, and exacerbations of chronic respiratory diseases like asthma (Reichardt et al., 2021). Furthermore, corticosteroids effectively manage acute respiratory distress syndrome (ARDS) (Chang et al., 2022).

Despite their clinical utility, prolonged use of glucocorticoids is associated with significant adverse effects. Dexamethasone, in particular, has been linked to metabolic disturbances such as hyperglycemia, insulin resistance, hepatic steatosis, and the development of type II diabetes (Madamsetty et al., 2022). Additionally, extended dexamethasone exposure has been shown to induce degenerative necrosis and inflammatory changes in hepatic tissues in experimental models (Ahmad, 2024).

Given the toxicity associated with prolonged glucocorticoid use, there is a growing interest in exploring dietary and botanical interventions to mitigate these adverse effects. Botanical drugs with antioxidant and anti-inflammatory properties are increasingly investigated as adjunctive therapies in metabolic and hepatic disorders. *Spirulina platensis*, a blue-green microalga classified as a botanical drug, has garnered attention due to its diverse pharmacological activities. It exhibits a unique spiral morphology and thrives in extreme environmental conditions. The species most commonly utilized in dietary and therapeutic applications are *Spirulina maxima* and *Spirulina platensis* (Althobaiti et al., 2024).

Spirulina platensis contains 65%–71% protein by dry weight, including 47% essential amino acids such as methionine. It also provides 15%–25% carbohydrates, 8%–13% minerals, 3%–7% lipids, and 8%–10% fiber. Moreover, it is a rich source of chlorophyll, phycocyanin, carotenoids, vitamins, and various bioactive metabolites (Iyer, 2011). Siva Kiran et al. (2015) showed that the blue-green algae extract Spirulina platensis is used worldwide as a meal and botanical drug. It is a healthy botanical drug with amino acids, lipids, vitamins, minerals, and antioxidants. A recent study has connected Spirulina platensis to immunomodulatory, pro-inflammatory, antioxidant, anti-cancer, and antiviral effects in animal models and humans. Hypolipidemic, hypoglycemic, and hypotensive effects are emerging, as are other health benefits, although hepatic consequences are poorly studied (Reboleira, 2019). *Spirulina platensis* possesses a substantial concentration of macro and micronutrients, necessary amino acids, proteins, lipids, vitamins, minerals, and antioxidants. It has a considerable vitamin B12 concentration and is an excellent source of beta-carotene, iron, calcium, and phosphorus. One gram of spirulina protein is equivalent to one pound of assorted vegetables. Spirulina enhances various human health parameters, from addressing malnutrition to providing antioxidant metabolites (Sonawane et al., 2023). This study was designed to evaluate the hepatoprotective potential of *Spirulina platensis* aqueous extract (SPAE) against DEX-induced oxidative stress, apoptosis, lipid disturbances, and transcriptional dysregulation in male rats. The underlying mechanisms, including modulation of redox-sensitive signaling pathways (e.g., Nrf2, AMPK) and apoptotic regulators (Bax, Bcl-2, p53), were also investigated.

## 2 Materials and methods

### 2.1 Botanical drug authentication and aqueous extraction

The powdered biomass used, *Spirulina platensis* (Product Number 741094) was bought from Sigma-Aldrich Chemicals Co., St. Louis, MO, United States. Taxonomic authentication confirmed the identity as *Spirulina platensis* (Gomont) Geitler, belonging to the family Oscillatoriaceae. A voucher specimen (Specimen ID: SP-DEX2025) has been deposited at the Herbarium of the Department of Botany, Faculty of Science, [Kafrelsheikh University, Egypt].

The extract was prepared by suspending 125 g of Spirulina powder in 1,000 mL of distilled water, followed by 24-h agitation at 30 °C. The suspension was filtered, centrifuged (5,000 rpm, 10 min), and concentrated under reduced pressure at 35 °C. The final yield was 25 g, yielding a drug–extract ratio (DER) of 5:1 (w/w). The extract was stored at 4 °C (Chu et al., 2010). See the HPLC analysis (Supplementary two and Phenolic profile of *Spirulina platensis* Supplementary Table S2).

### 2.2 Experimental design

Forty male Sprague Dawley rats (150 ± 10 g) were acclimatized for 1 week with free access to standard feed and water under sanitary conditions (Kasem et al., 2025).

After this week, the rats were arbitrarily allocated into four groups, each including 10 rats. Group 1 (Control): Received standard diet and saline solution. Group 2 (DEX): Received intraperitoneal DEX (10 mg/kg) on alternate days for 28 days (Mada, 2019). Group 3 (DEX + SPAE 400): Received DEX (10 mg/kg, I/P) and SPAE orally at 400 mg/kg/day. Group 4 (DEX + SPAE 800): Received DEX (10 mg/kg, I/P) and SPAE orally at 800 mg/kg/day (Abdelmaksoud and Abdeen, 2019).

The 400 mg/kg and 800 mg/kg body weight/day doses for *Spirulina platensis* aqueous extract were selected based on previous studies demonstrating safety and hepatoprotective efficacy in rodent models (Abdelmaksoud and Abdeen, 2019; Ghallab et al., 2025), respectively.

Twenty-four hours after the final treatment following the 28-day study, delivery was conducted under ketamine/xylazine anaesthesia at dosages of 7.5 and 1.0 mg/kg by intraperitoneal infusion (El-Aarag et al., 2017). Blood specimens were collected in dry centrifuge tubes from the hepatic portal vein and centrifuged for 10 min at 4,000 rpm. The serum was stored in sterile vials at −20 °C until analysis. The liver was exercised, weighed, and utilized for subsequent study.

**FIGURE 1:**
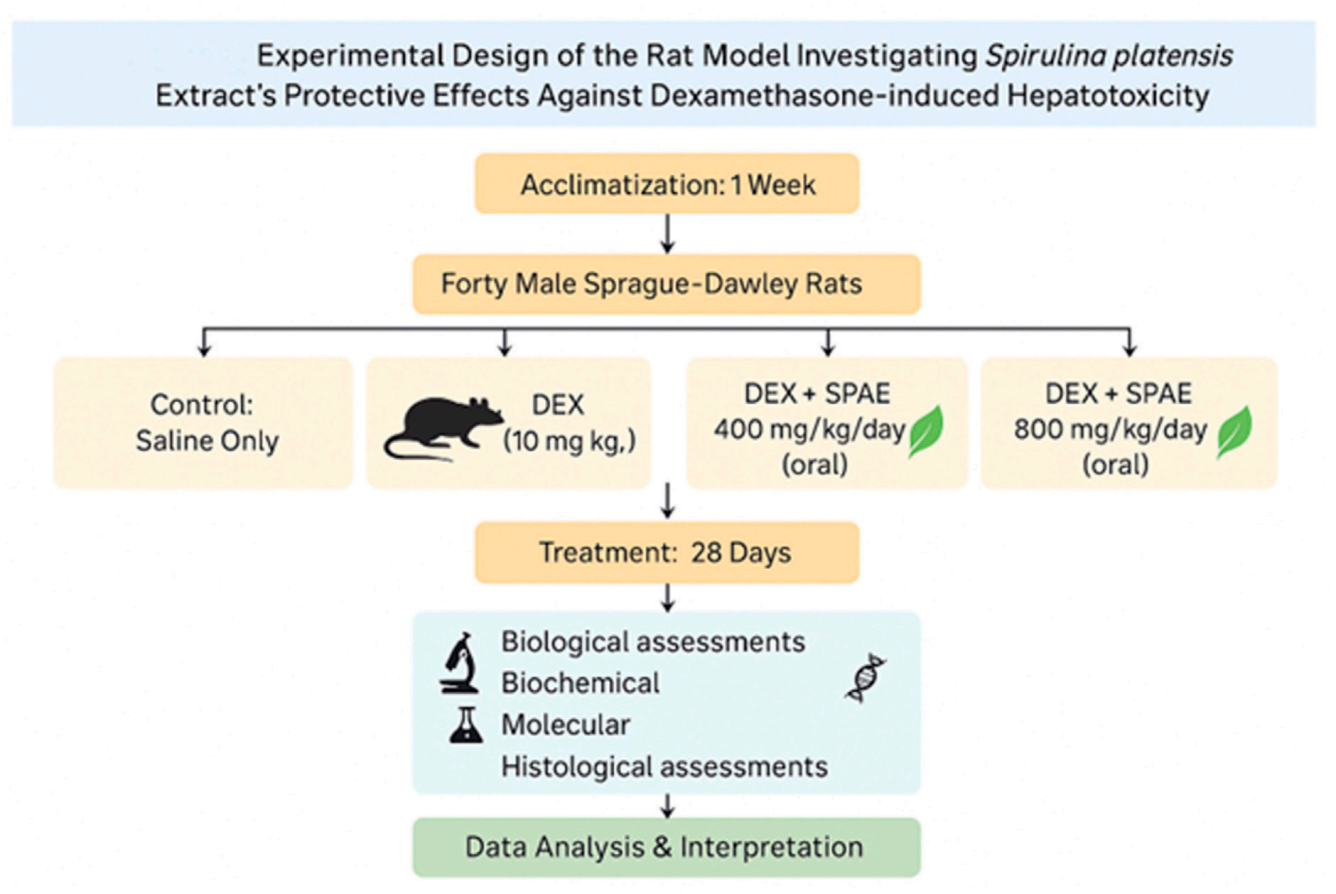
Experimental design of animal study.

The liver specimens were preserved in a 10% formalin saline solution for histological analysis. Additional samples were stored at −20 °C to prepare tissue homogenates to assess antioxidant properties. The homogenate underwent centrifugation at 10,000 rpm for 20 min. The supernatant was utilized for the assessment of several laboratory analyses (see Figure 1).

### 2.3 Biological and nutritional parameters

At the end of the experiment, feed intake, weight gain, liver weight to body weight, and feed efficiency ratio were calculated following (Hafez Hafez et al., 2025).

### 2.4 Biochemical analysis of serum

The following was used to calculate (LDL-C) and (VLDL-C): Bio-diagnostic Company, Egypt, for total serum cholesterol (TC), triglycerides (TG), and high-density lipoprotein cholesterol (HDL-C) (Friedewald et al., 1972). Following the guidelines supplied by Diamond Diagnostics (Egypt), the levels of hepatocellular enzymes (AST, ALT, and ALP) were measured in the serum. The method outlined in the reference was used for the AST and ALT evaluation (Reitman and Frankel, 1957), while ALP was measured as outlined in Belfield and Goldberg (1971). γ-Glutamyl transferase (GGT) was assessed following (Szasz, 1969). Serum total bilirubin and direct bilirubin were estimated using kits purchased from Biodiagnostics (Giza, Egypt) following (Walters and Gerarde, 1970). Similarly, total blood protein, albumin, and globulin levels were determined in Doumas et al. (1981), Doumas et al. (1971), and Watson (1966), respectively.

### 2.5 Liver tissue oxidant/antioxidant activity evaluation

Following the protocol laid out earlier, the liver homogenate was produced (Kasim, 2025). Following the method outlined by Ohkawa et al., oxidative injury biomarkers such as lipid peroxidation biomarkers like (MDA) and (NO) were assessed in the liver homogenate (Ohkawa et al., 1979) and (Koltuksuz et al., 2000), respectively. Also examined were levels of (GPx), (GSH), and (SOD), following the methods outlined earlier (Beutler et al., 1963; Paglia and Valentine, 1967; Nishikimi et al., 1972), respectively.

### 2.6 Comet assay (DNA damage evaluation)

One gram of crushed liver tissue was suspended in 1 mL of ice-cold PBS. The mixture was stirred for 5 min and then passed through a filter. From the resulting cell suspension, 100 µL was blended with 600 µL of 0.8% low-melting agarose prepared in PBS. Then, 100 µL of this combination was evenly spread onto microscope slides, which were immersed in a lysis solution made of NaCl (2.5 M), EDTA (100 mM), TRIS buffer (10 mM), 1% lauryl sarcosinate, 1% Triton X-100, and 10% DMSO for 15 min. The treated slides were placed in an electrophoresis chamber containing the same buffer without SDS. Electrophoresis was performed at 2 V/cm and 100 mA for 2 min. Afterwards, 80 µL of ethidium bromide (2%–7%) was applied, followed by a 20-min rinse with water. Slides were then stained with GelRed for 10 min. DNA migration patterns were observed using a fluorescent microscope at ×40 magnification and an excitation filter ranging from 420 to 490 nm. Image capture and analysis were done using the Komet-5 software (Kinetic Imaging Ltd., Liverpool, United Kingdom) connected to a CCD camera (Olive and Banáth, 2006).

### 2.7 Histopathological examination

Preparation for paraffin fixation included removing the liver and fixing it in a 10% neutral buffered formaldehyde solution. Following this, the liver was washed with xylol. Hematoxylin and eosin were used to stain a 4–5 µm-thick section for histological examination (H&E) (Dawoud et al., 2021).

### 2.8 Immunohistochemical staining

Following the protocol, immunohistochemical labelling of specific liver sections from both the control and treatment groups was conducted to assess caspase-3 as a marker of apoptosis using avidin-biotin-peroxidase (DAB, Sigma Chemical Co.) (Koite et al., 2022). A monoclonal antibody to α-SMA and caspase-3 was used to incubate tissue sections, and the expression of these proteins was detected using the chromogen 3,3-diaminobenzidine tetrahydrochloride (DAB, Sigma-Aldrich^®^) stain.

### 2.9 RT-PCR

Gene expression analysis in the hepatic tissue was performed using quantitative real-time PCR (qRT-PCR). Total RNA was isolated from approximately 100 mg of brain tissue utilizing TRIzol reagent (Invitrogen, Carlsbad, CA, United States), and RNA concentration and purity were determined using a NanoDrop spectrophotometer. Only RNA samples with an A260/A280 ratio ≥1.8 were considered suitable for downstream applications. First-strand complementary DNA (cDNA) synthesis was conducted using a cDNA synthesis kit (Fermentas, Waltham, MA, United States). Specific primers and SYBR Green Master Mix used for amplifying the target genes are detailed in Supplementary Table S1. Gene expression was normalized against the internal control gene *GAPDH* (glyceraldehyde 3-phosphate dehydrogenase). Relative expression levels were calculated using the 2^(-ΔΔCt) method (Livak and Schmittgen, 2001).

### 2.10 Statistical analysis

Data are expressed as mean ± standard deviation (SD). Statistical analyses were performed using SPSS version 26. Differences between groups were assessed using one-way ANOVA followed by Duncan’s Multiple Range Test. Statistical significance was considered at *p* < 0.05.

## 3 Results

### 3.1 Effect of SPAE on dietary parameters and liver weight

The administration of dexamethasone (DEX) significantly impaired dietary performance, as evidenced by a marked reduction in feed intake (FI: 17.67 ± 2.26 g/day) and body weight gain (BWG: 19.50 ± 3.39 g) compared to the negative control group (FI: 34.03 ± 2.05 g/day; BWG: 59.17 ± 4.83 g; *p* < 0.05).

Co-treatment with *Spirulina platensis* aqueous extract (SPAE) at 400 mg/kg and 800 mg/kg significantly improved these parameters relative to the DEX group. At 400 mg/kg, SPAE increased FI to 25.54 ± 1.96 g/day and BWG to 41.33 ± 2.58 g (*p* < 0.05 vs. DEX). The 800 mg/kg dose further enhanced FI to 29.66 ± 0.92 g/day and BWG to 48.17 ± 2.93 g (*p* < 0.05 vs. DEX and 400 mg/kg), indicating a dose-dependent protective effect that nearly restored values to those of the control.

The feed efficiency ratio (FER) remained statistically unchanged among all groups, ranging from 0.0358 to 0.0360 (*p* > 0.05), suggesting that energy conversion efficiency was not significantly affected.

In terms of liver weight, DEX administration led to hepatomegaly, with a significant increase in liver mass (11.38 ± 1.12 g) compared to the control group (7.34 ± 0.38 g; *p* < 0.05). SPAE at 400 mg/kg reduced liver weight to 9.29 ± 0.78 g (*p* < 0.05 vs. DEX), while the 800 mg/kg dose produced a more pronounced effect, lowering liver weight to 8.10 ± 0.27 g (*p* < 0.05 vs. DEX and 400 mg/kg). These results suggest that SPAE attenuates DEX-induced hepatomegaly in a dose-responsive manner, as shown in Table 1.

**TABLE 1:** Effect of SPAE on dietary evaluation and liver weight in rats administered dexamethasone.

Groups	FI (g/day)	BWG (g)	FER	Liver weight
Negative control	34.03 ± 2.05^a^	59.17 ± 4.83^a^	0.0360 ± 0.00245^a^	7.34 ± 0.38^d^
DEX (10 g/kg)	17.67 ± 2.26 ^d^	19.50 ± 3.39 ^d^	0.0358 ± 0.00041^a^	11.38 ± 1.12^a^
DEX + Spirulina (400 mg/kg)	25.54 ± 1.96^c^	41.33 ± 2.58^c^	0.0360 ± 0.00041^a^	9.29 ± 0.78^b^
DEX + Spirulina (800 mg/kg)	29.66 ± 0.92 ^b^	48.17 ± 2.93 ^b^	0.0358 ± 0.00041^a^	8.10 ± 0.27^c^

Data expressed as (mean ± SD, *n* = 6).

^a,b,c,d^Mean values in the same column with completely different letters are significantly different at p < 0.05.

### 3.2 SPAE improves lipid profile in DEX-treated rats

Dexamethasone (DEX) administration caused profound dysregulation of lipid metabolism. Compared to the negative control group, DEX-treated rats exhibited a significant elevation in serum total cholesterol (TC: 209.67 ± 6.44 mg/dL vs. 76.16 ± 2.42 mg/dL), triglycerides (TG: 291.25 ± 2.16 mg/dL vs. 67.62 ± 2.43 mg/dL), low-density lipoprotein (LDL: 128.36 ± 7.01 mg/dL vs. 12.61 ± 1.87 mg/dL), and very low-density lipoprotein (VLDL: 58.25 ± 0.43 mg/dL vs. 13.52 ± 0.49 mg/dL) (*p* < 0.05 for all). Concurrently, there was a marked reduction in high-density lipoprotein (HDL: 23.07 ± 1.75 mg/dL vs. 49.71 ± 1.99 mg/dL; *p* < 0.05).

SPAE treatment significantly ameliorated these lipid abnormalities in a dose-dependent manner. At 400 mg/kg, SPAE reduced TC to 139.38 ± 2.37 mg/dL, TG to 181.41 ± 3.96 mg/dL, LDL to 69.99 ± 1.84 mg/dL, and VLDL to 36.26 ± 0.81 mg/dL, while increasing HDL to 33.13 ± 3.23 mg/dL (*p* < 0.05 vs. DEX for all parameters).

The higher dose of SPAE (800 mg/kg) produced a more pronounced normalization of lipid levels: TC decreased to 103.34 ± 2.70 mg/dL, TG to 111.33 ± 4.65 mg/dL, LDL to 37.04 ± 4.59 mg/dL, and VLDL to 22.26 ± 0.93 mg/dL, while HDL increased to 43.65 ± 2.64 mg/dL. These improvements were statistically significant compared to the DEX and 400 mg/kg groups (*p* < 0.05), and lipid levels in the 800 mg/kg group closely approximated those of the control group, as shown in Table 2.

**TABLE 2:** Effect of SPAE on lipid profile in serum of rats administrated dexamethasone (mean ± SD, *n* = 6).

	Negative control	DEX (10 mg/kg)	DEX + spirulina (400 mg/kg)	DEX + spirulina (800 mg/kg)
TC (mg/dL)	76.16 ± 2.42 ^d^	209.67 ± 6.44^a^	139.38 ± 2.37^b^	103.34 ± 2.70^c^
TG (mg/dL)	67.62 ± 2.43^d^	291.25 ± 2.16^a^	181.41 ± 3.96^b^	111.33 ± 4.65^c^
HDL (mg/dL)	49.71 ± 1.99^a^	23.07 ± 1.75^d^	33.13 ± 3.23^c^	43.65 ± 2.64^b^
LDL (mg/dL)	12.61 ± 1.87^d^	128.36 ± 7.01^a^	69.99 ± 1.84^b^	37.04 ± 4.59^c^
VLDL (mg/dL)	13.52 ± 0.49 ^d^	58.25 ± 0.43^a^	36.26 ± 0.81 ^b^	22.26 ± 0.93^c^

Data expressed as (mean ± SD, *n* = 6).

^a,b,c,d^Mean values in the same row with completely different letters are significantly different at p < 0.05.

These results confirm that SPAE exerts a protective, dose-dependent hypolipidemic effect against DEX-induced dyslipidemia.

### 3.3 SPAE restores liver function markers

DEX administration caused significant hepatotoxicity, as indicated by elevated serum levels of key liver enzymes. Aspartate aminotransferase (AST), alanine aminotransferase (ALT), and alkaline phosphatase (ALP) were significantly increased in the DEX group (AST: 319.33 ± 3.95 U/L; ALT: 153.46 ± 5.38 U/L; ALP: 611.50 ± 7.36 U/L) compared to the negative control group (AST: 123.41 ± 2.61 U/L; ALT: 45.17 ± 3.24 U/L; ALP: 332.56 ± 5.00 U/L; *p* < 0.05 for all).

SPAE treatment significantly reduced these elevated enzyme levels in a dose-dependent manner. At 400 mg/kg, SPAE lowered AST to 267.94 ± 2.62 U/L, ALT to 100.88 ± 3.23 U/L, and ALP to 463.60 ± 11.68 U/L (*p* < 0.05 vs. DEX). The 800 mg/kg dose yielded further improvements (AST: 201.79 ± 4.72 U/L; ALT: 73.75 ± 2.65 U/L; ALP: 392.57 ± 3.20 U/L), with enzyme levels approaching those of the control group (*p* < 0.05 vs. DEX and 400 mg/kg).

Gamma-glutamyl transferase (GGT), total bilirubin (T.BIL), and direct bilirubin (D.BIL) were also markedly elevated in the DEX group (GGT: 21.37 ± 0.77 U/L; T.BIL: 1.06 ± 0.06 mg/dL; D.BIL: 0.63 ± 0.07 mg/dL) relative to controls (GGT: 8.23 ± 0.80 U/L; T.BIL: 0.26 ± 0.02 mg/dL; D.BIL: 0.17 ± 0.01 mg/dL; *p* < 0.05). SPAE at 400 mg/kg significantly reduced these levels (GGT: 15.05 ± 1.61 U/L; T.BIL: 0.50 ± 0.07 mg/dL; D.BIL: 0.39 ± 0.06 mg/dL), with greater reductions seen at 800 mg/kg (GGT: 11.15 ± 0.37 U/L; T.BIL: 0.44 ± 0.08 mg/dL; D.BIL: 0.31 ± 0.05 mg/dL; *p* < 0.05 vs. DEX), indicating restoration toward normal hepatic excretory function.

Moreover, DEX significantly reduced serum total protein (TP), albumin (ALB), and globulin (GLB) levels (TP: 4.70 ± 0.49 g/dL; ALB: 2.84 ± 0.93 g/dL; GLB: 2.03 ± 0.11 g/dL) when compared to the control group (TP: 7.86 ± 0.12 g/dL; ALB: 4.51 ± 0.06 g/dL; GLB: 3.35 ± 0.06 g/dL; *p* < 0.05). SPAE at 400 mg/kg significantly restored TP (6.94 ± 0.18 g/dL), ALB (4.24 ± 0.18 g/dL), and GLB (2.70 ± 0.04 g/dL), while the 800 mg/kg dose further normalized TP (7.57 ± 0.14 g/dL), ALB (4.60 ± 0.18 g/dL), and GLB (2.97 ± 0.04 g/dL), with most values statistically indistinguishable from the control group (*p* > 0.05) as shown in Table 3.

**TABLE 3:** Effect of SPAE on liver functions of rats administered dexamethasone (mean ± SD, *n* = 6).

Groups	Negative control	DEX (10 mg/kg)	DEX + spirulina (400 mg/kg)	DEX + spirulina (800 mg/kg)
AST (U/L)	123.41 ± 2.61^d^	319.33 ± 3.95^a^	267.94 ± 2.62^b^	201.79 ± 4.72^c^
ALT (U/L)	45.17 ± 3.24^d^	153.46 ± 5.38^a^	100.88 ± 3.23^b^	73.75 ± 2.65^c^
ALP (U/L)	332.56 ± 5.00 ^d^	611.50 ± 7.36^a^	463.60 ± 11.68 ^b^	392.57 ± 3.20^c^
GGT (U/L)	8.23 ± 0.80^d^	21.37 ± 0.77^a^	15.05 ± 1.61^b^	11.15 ± 0.37^c^
T. BIL (mg/dL)	0.26 ± 0.02^c^	1.06 ± 0.06^a^	0.50 ± 0.07^b^	0.44 ± 0.08^b^
D.BIL (mg/dL)	0.17 ± 0.01^d^	0.63 ± 0.07^a^	0.39 ± 0.06^b^	0.31 ± 0.05^c^
T.P (g/dL)	7.86 ± 0.12^a^	4.70 ± 0.49^c^	6.94 ± 0.18^b^	7.57 ± 0.14^a^
ALB (g/dL)	4.51 ± 0.06^a^	2.84 ± 0.93^b^	4.24 ± 0.18^a^	4.60 ± 0.18^a^
GLB (g/dL)	3.35 ± 0.06^a^	2.03 ± 0.11^d^	2.70 ± 0.04^c^	2.97 ± 0.04^b^

Data expressed as (mean ± SD, *n* = 6).

^a,b,c,d^Mean values in the same row with completely different letters are significantly different at p < 0.05.

These findings confirm that SPAE provides a dose-dependent hepatoprotective effect, mitigating DEX-induced hepatic dysfunction and restoring biochemical liver integrity.

### 3.4 SPAE enhances antioxidant defense and reduces oxidative stress

**FIGURE 2:**
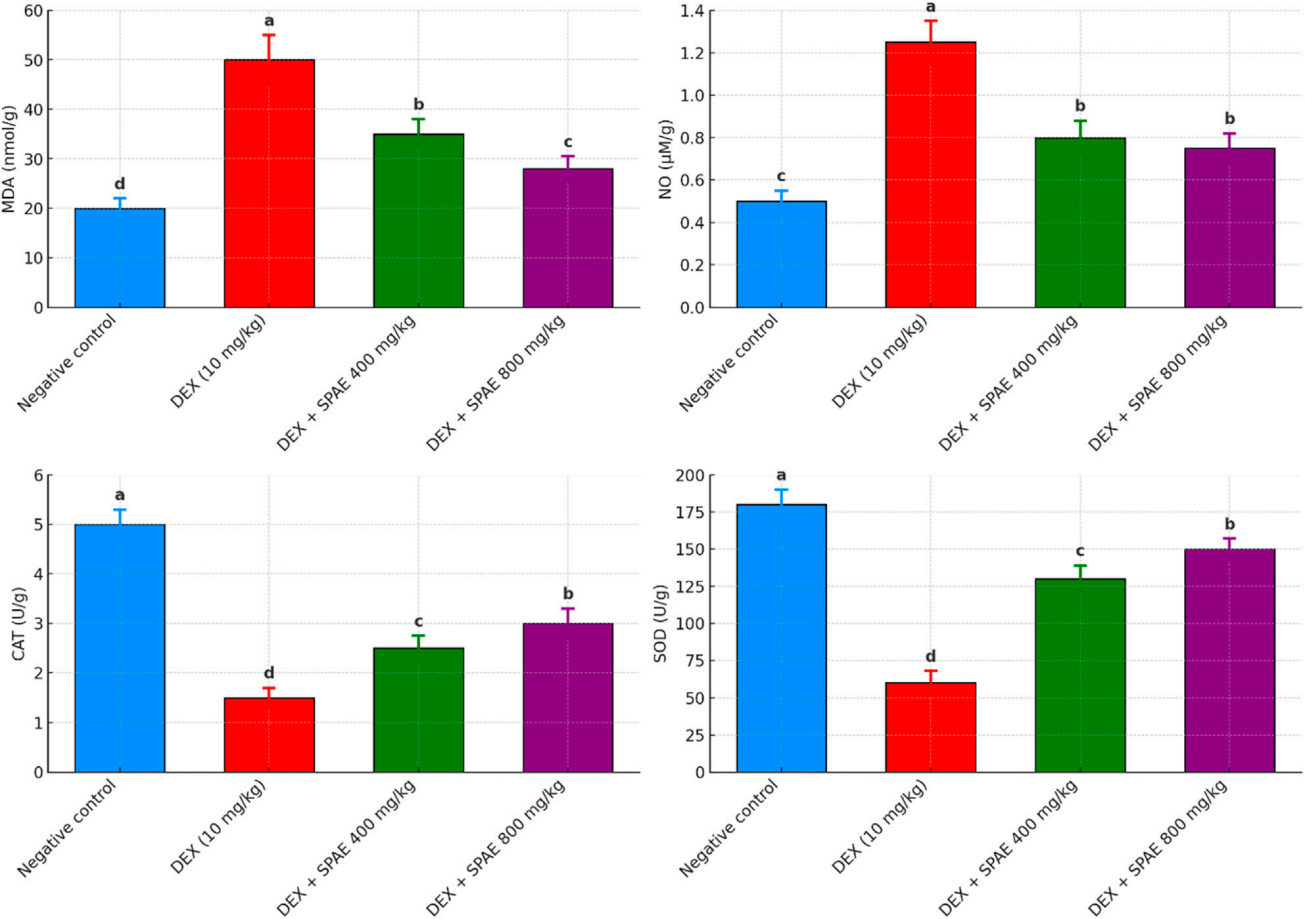
Effects of SPAE on MDA, NO, CAT, and SOD in liver tissues of rats administered Dexmethasone. Data expressed as (mean ± SD, *n* = 6). ^a,b,c,d^Mean values in the same column with completely different letters are significantly different at p < 0.05.

The effect of SPAE and DEX on lipid peroxidation marker (MDA& NO) levels and antioxidant enzyme (CAT & SOD) activity in rat liver tissues. The DEX group showed an improvement in (MDA & NO) and a decline in (CAT & SOD) compared to the negative control group after DEX injection. While the DEX group showed no change in (MDA & NO), animals given SPAE (400 & 800 mg/kg) showed a considerable improvement in (CAT & SOD). The administrative group with SPAE (800 mg/kg) had the greatest results for MDA, CAT, and SOD, which is close to the normal group (as indicated in Figure 2).

### 3.5 SPAE attenuates genotoxicity induced by DEX

Dexamethasone (DEX) administration caused marked genotoxic damage in liver cells, as assessed by comet assay parameters, including tail length, tail DNA percentage, and tail moment. Rats in the DEX group showed a significant increase in tail length (10.60 ± 0.36 µm), tail DNA % (13.6%), and tail moment (73.27 ± 1.10), compared to the control group (tail length: 1.60 ± 0.08 µm; tail DNA %: 3.1%; tail moment: 3.78 ± 0.20; *p* < 0.05 for all), indicating extensive DNA strand breaks.

Treatment with *Spirulina platensis* aqueous extract (SPAE) significantly attenuated this DNA damage in a dose-dependent manner. At 400 mg/kg, SPAE reduced tail length to 4.59 ± 0.37 µm, tail DNA% to 8.69%, and tail moment to 16.61 ± 0.54 (*p* < 0.05 vs. DEX). The higher 400 mg/kg dose produced a more substantial protective effect, further reducing tail length to 2.62 ± 0.54 µm, tail DNA% % to 4.1%, and tail moment to 9.68 ± 0.28 (*p* < 0.05 vs. DEX and 400 mg/kg).

Additionally, the percentage of tailed cells decreased from 12% in the DEX group to 8% and 4% in the 400 mg/kg and 800 mg/kg Spirulina groups, respectively, while the percentage of untailed cells increased from 88% (DEX) to 92% and 96%.

**FIGURE 3:**
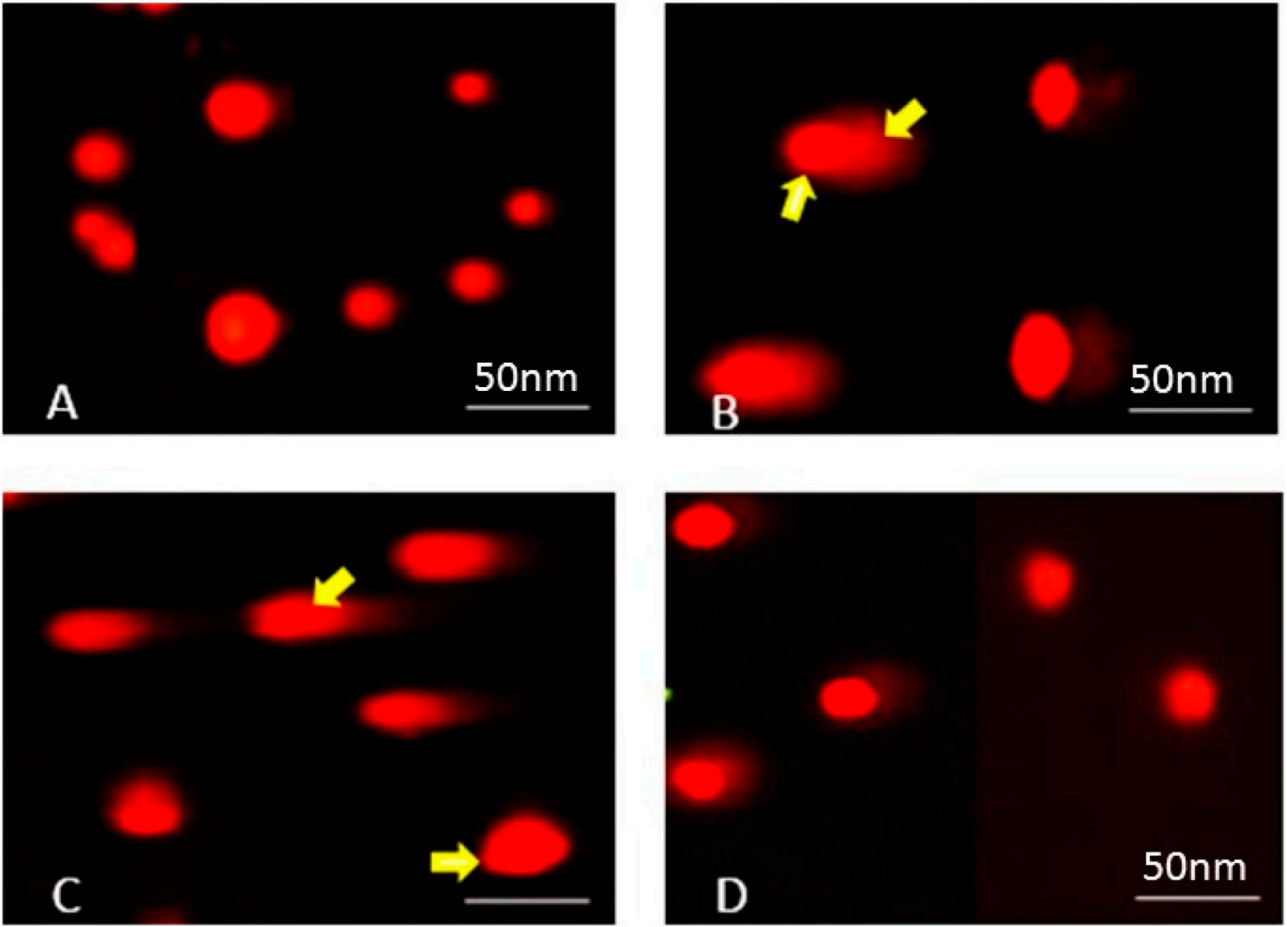
Photomicrographs representing DNA damage, using the comet assay, in rats of **(A)** control, **(B)** dexamethasone, **(C)** dexamethasone with spirulina 400, and **(D)** dexamethasone with spirulina 800.

These results demonstrate that SPAE effectively reduces DEX-induced DNA damage in hepatic tissues, with the 800 mg/kg dose offering greater genoprotective effects than 400 mg/kg (Table 4; Figure 3).

**TABLE 4:** Comet parameters in rat liver treated with dexamethasone and spirulina.

Groups	Tailed %	Untailed %	Tail length (µm)	Tail DNA %	Tail moment
Control	3	97	1.60 ± 0.08^d^	3.1	3.78 ± 0.20^d^
Dexamethasone	12	88	10.60 ± 0.36^a^	13.6	73.27 ± 1.10^a^
Spirulina 400	8	92	4.59 ± 0.37^b^	8.69	16.61 ± 0.54^b^
Spirulina 800	4	96	2.62 ± 0.54^c^	4.1	9.68 ± 0.28^c^

Data expressed as (mean ± SD, *n* = 6).

^a,b,c,d^Mean values in the same column with completely different letters are significantly different at p < 0.05.

### 3.6 Histopathological evaluation of liver tissues

Histological examination of liver sections stained with hematoxylin and eosin (H&E) revealed substantial architectural differences among the groups (Figure 3).

**FIGURE 4:**
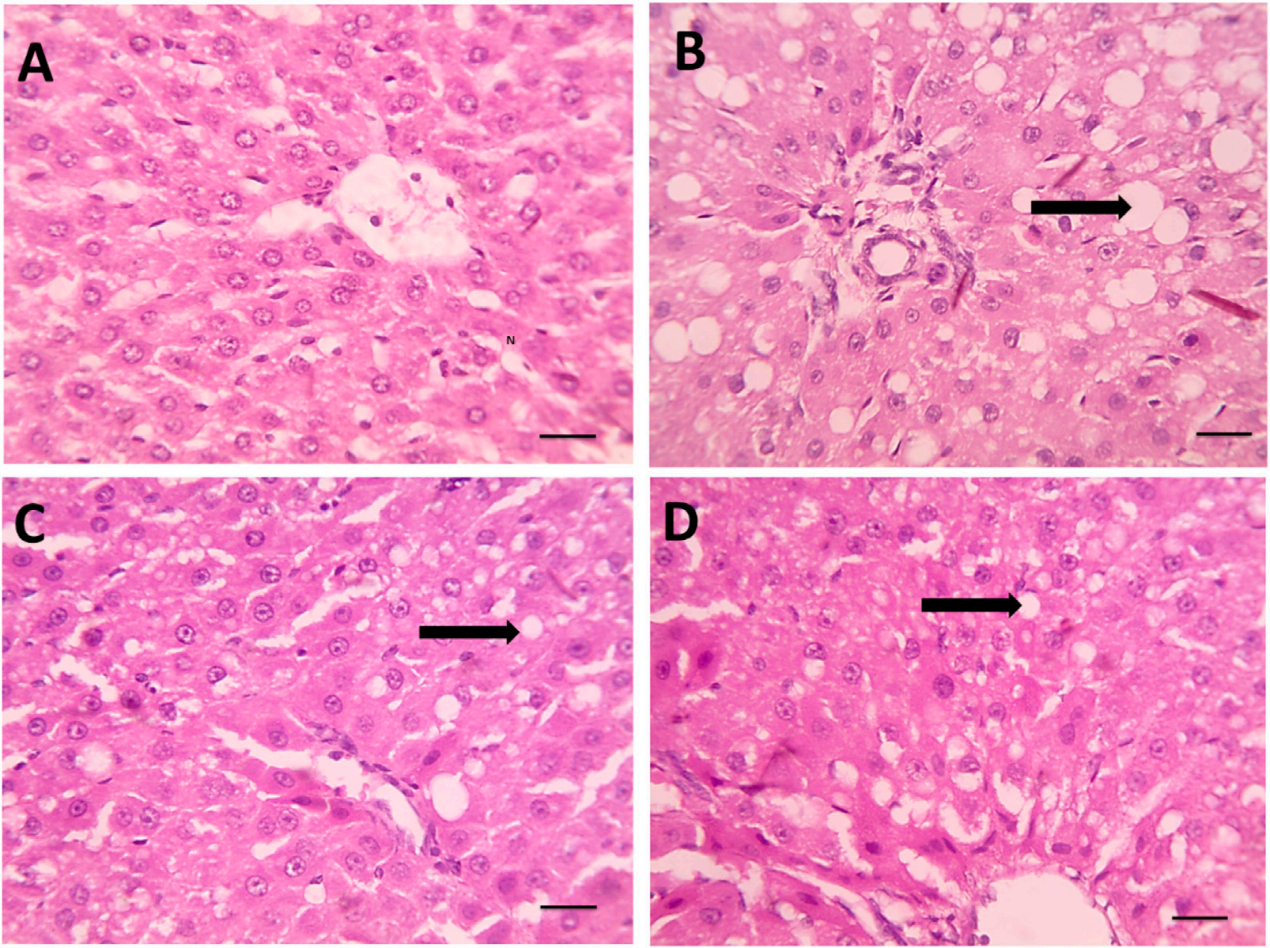
**(A)** Microscopic pictures of HE stained hepatic sections showing regular arrangement of hepatic cords around central veins with normal portal areas and sinusoids in the control group. **(B)** Hepatic sections from the Dexa group show macrovesicular steatosis, which is characterized by many large-sized fat vacuoles (black arrows). **(C)** Smaller and fewer fat vacuoles are seen in some hepatocytes in the treated group. **(D)** Spirulina 400, microvesicular steatosis characterized by a few small-sized fat vacuoles (black arrows) are seen in a few hepatocytes in the treated group, Spirulina 800 high magnification X: 400. Scale bar = 50 μm.

In the negative control group (Figure 4A), the liver displayed a normal hepatic architecture characterized by well-organized hepatic cords, intact central veins, and uniformly sized hepatocytes with prominent nuclei and eosinophilic cytoplasm. No signs of lipid accumulation, inflammation, or necrosis were observed.

Conversely, DEX-treated rats (Figure 4B) exhibited pronounced hepatic injury. Histological sections showed severe hepatocellular degeneration, extensive macrovesicular steatosis with large fat vacuoles (black arrow), cytoplasmic rarefaction, and sinusoidal dilatation. Disruption of hepatic cords and focal areas of necrosis were also evident, confirming the hepatotoxic effect of DEX.

Rats treated with SPAE at 800 mg/kg (Figure 4C) showed moderate histological improvement. Fatty degeneration was present but substantially reduced, with fewer and smaller lipid droplets than the DEX group. The hepatic cords appeared more organized, and inflammation was notably less severe.

The 800 mg/kg SPAE group (Figure 4D) demonstrated marked histological restoration. Hepatocytes exhibited nearly normal morphology, with only mild vacuolization (black arrow) and minimal fatty change. The sinusoidal spaces and central vein regions were largely preserved, closely resembling the architecture observed in control liver tissues.

These observations suggest that SPAE confers a dose-dependent protective effect against DEX-induced hepatic steatosis and structural liver damage.

### 3.7 Caspase-3 expression via immunohistochemistry

**FIGURE 5:**
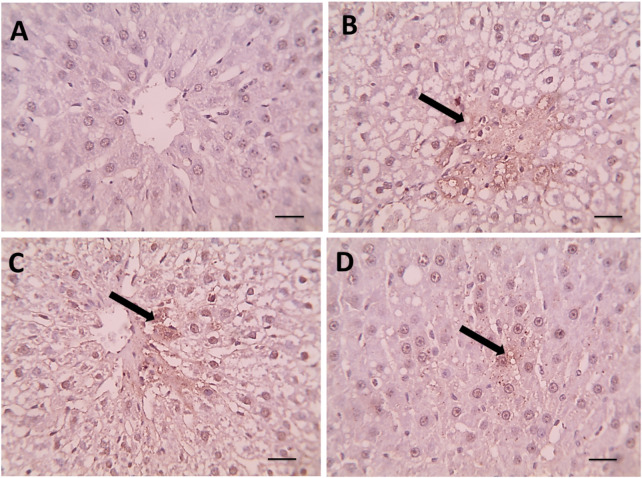
**(A)** Microscopic pictures of immunostained hepatic sections against caspase showing negative staining in hepatocytes in the control group. Hepatic sections from the **(B)** Dexa group showing positive brown staining of some degenerated hepatocytes (black arrows). **(C)** Hepatic sections from the treated group Spirulina 400 showed mild positive brown staining of a few degenerated hepatocytes (black arrows). **(D)** Hepatic sections from the treated group Spirulina 800 showing very mild positive brown staining of individual hepatocytes (black arrow). Magnification X: 400. Scale bar = 50 μm.

**FIGURE 6:**
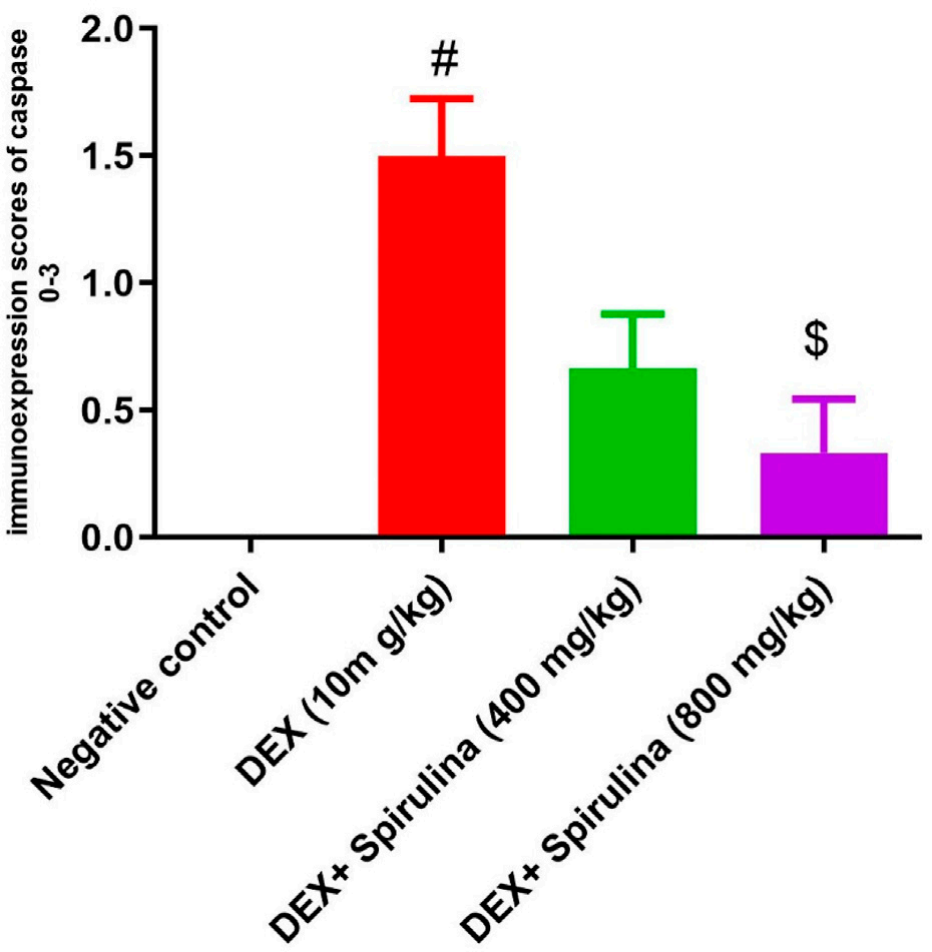
Bars represent scores of caspase immunoexpression in hepatocytes in pictures picked up at magnification X: 400. A significant decrease of caspase immunoexpression is seen in the treated group Spirulina 400, compared to the Dexa group. # means significantly different from the control group when P < 0.01, and $ means substantially different from the Dexa group when P < 0.05. Data analyzed by Kruskal–Wallis, followed by Dunn’s test to compare all means.

The control and spirulina-treated rats (400) had comparable low expression of caspase-3 in their liver tissue, indicated by the lack of positive brown staining (Figures 5A,D). The liver of DEX-treated rats exhibited varying levels of positive caspase-3 expression, marked by brown-stained nuclei of apoptotic cells (Figure 5B). Conversely, spirulina treatment in rats subjected to Dex demonstrated significant improvement in the elevated count of positively expressed apoptotic cells as seen by reduced or absent caspase-3 expression. (Figures 5C,D). The highest caspase immunoexpressing score was given to the Dexa group, and the score of the spirulina group was significantly lower (Figure 6).

### 3.8 Gene expression modulation by SPAE

**FIGURE 7:**
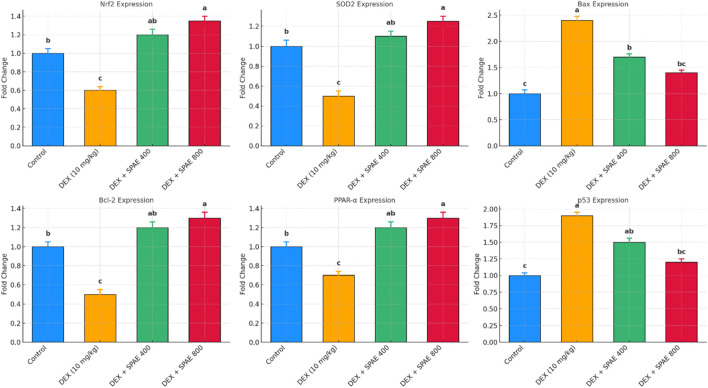
The bar graphs illustrate the relative mRNA expression levels (fold change) of key genes: Nrf2, SOD2 (oxidative stress markers), Bax, Bcl-2, p53 (apoptosis-related genes), and PPAR-α (lipid metabolism regulator) in the liver tissue of male rats across four experimental groups: Control, DEX (10 mg/kg), DEX + *Spirulina* 400 mg/kg, and DEX + *Spirulina* 800 mg/kg. Gene expression was assessed using quantitative RT-PCR and normalized to the reference gene β-actin. Data are presented as mean ± SD (*n* = 6). Different superscript letters indicate statistically significant differences between groups (*p* < 0.05).

The mRNA expression levels of oxidative stress, apoptosis, lipid metabolism, and DNA damage-related genes were evaluated using quantitative RT-PCR. The results demonstrated significant alterations induced by dexamethasone (DEX), which were notably modulated by *Spirulina platensis* co-treatment. DEX significantly downregulated Nrf2 expression compared to the control group (*p* < 0.05). Co-administration of Spirulina at 400 mg/kg and 800 mg/kg doses restored Nrf2 expression, with the 800 mg/kg dose showing a significant upregulation above control levels (*p* < 0.05). This suggests activation of the antioxidant defence system by Spirulina. A similar trend was observed in SOD2 expression. DEX markedly suppressed SOD2 levels (*p* < 0.05), indicating oxidative stress. Spirulina treatment significantly reversed this suppression in a dose-dependent manner, with the higher dose leading to expression markedly greater than control (*p* < 0.05).DEX significantly elevated Bax mRNA expression (*p* < 0.05), suggesting enhanced pro-apoptotic activity. Spirulina at both doses attenuated this increase, with the 800 mg/kg group showing the most notable reduction (*p* < 0.05), indicating anti-apoptotic potential. In contrast, Bcl-2, an anti-apoptotic gene, was significantly downregulated in the DEX group (*p* < 0.05). Spirulina treatment, especially at 800 mg/kg, significantly restored Bcl-2 expression to levels exceeding the control (*p* < 0.05), further supporting the anti-apoptotic role of Spirulina.DEX caused a significant decrease in PPAR-α expression compared to control (*p* < 0.05), indicative of impaired lipid metabolism. Spirulina administration restored PPAR-α levels in a dose-responsive manner, with the 800 mg/kg group showing a significant increase above control (p < 0.05). p53, a key regulator of DNA damage and apoptosis, was significantly upregulated in the DEX group (p < 0.05). Spirulina treatment downregulated p53 expression, with 800 mg/kg considerably lowering it compared to DEX alone (p < 0.05), indicating reduced DNA damage or stress response, as shown in Figure 7.

**FIGURE 8:**
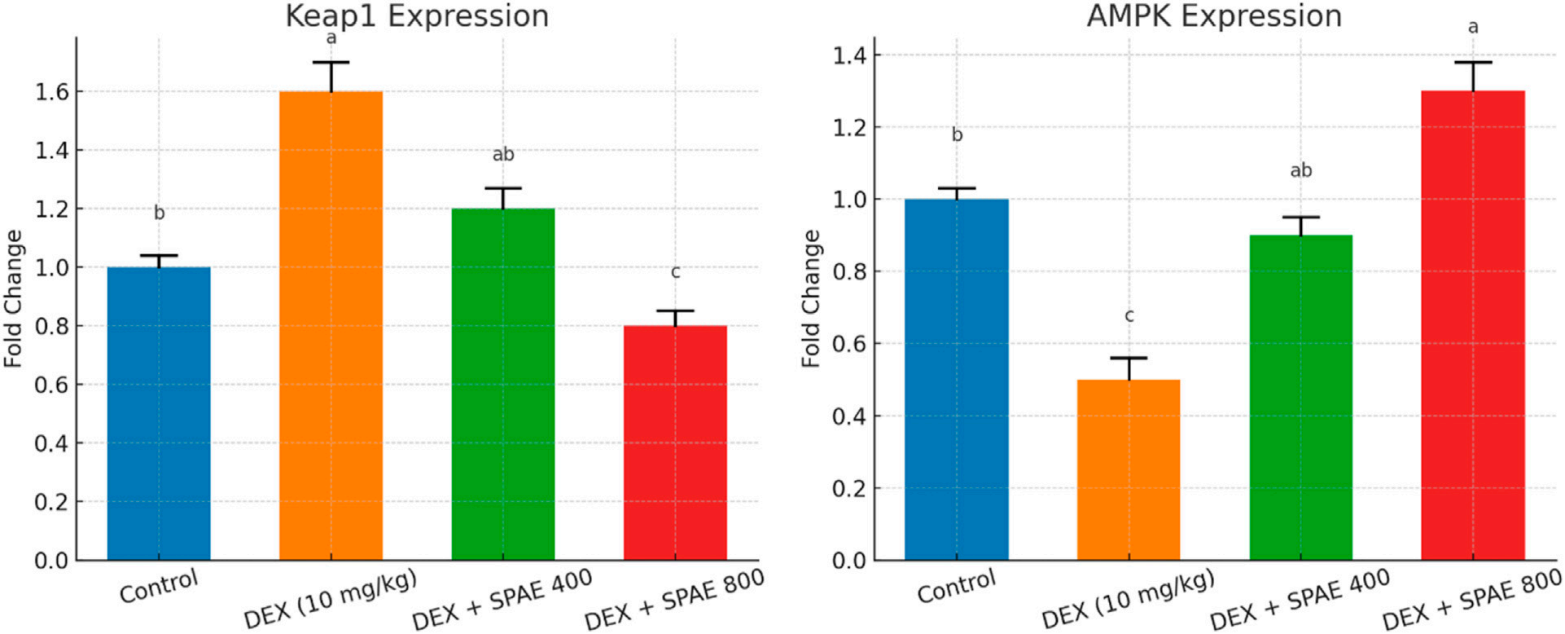
Effects of *Spirulina platensis* aqueous extract (SPAE) on hepatic *Keap1* gene and *AMPK* gene expression in dexamethasone (DEX)-treated rats. Data are expressed as mean ± SD (*n* = 6). Gene expression was assessed using quantitative RT-PCR and normalized to the reference gene β-actin. Data are presented as mean ± SD (*n* = 6). Different superscript letters indicate statistically significant differences between groups (*p* < 0.05).

Quantitative gene expression analysis revealed significant alterations in Keap1 and AMPK levels across the experimental groups. Dexamethasone (DEX) administration markedly increased Keap1 expression (1.60 ± 0.10) compared to the control group (1.00 ± 0.04), indicating suppression of the *Nrf2* pathway. Co-treatment with *Spirulina platensis* aqueous extract (SPAE) led to a dose-dependent reduction in Keap1 expression, with the 800 mg/kg dose showing the most pronounced effect (0.80 ± 0.05), significantly lower than both the DEX and SPAE 400 mg/kg groups (1.20 ± 0.07). Conversely, AMPK expression was significantly downregulated by DEX (0.50 ± 0.06) relative to control (1.00 ± 0.03). SPAE treatment restored AMPK levels in a dose-dependent manner, with the 800 mg/kg group showing significantly increased expression (1.30 ± 0.08), exceeding even the control levels. The SPAE 400 mg/kg group (0.90 ± 0.05) also showed significant improvement compared to DEX alone. These findings suggest that SPAE mitigates DEX-induced hepatic stress by modulating upstream regulators of antioxidant and metabolic pathways, namely, Keap1 and AMPK, as shown in Figure 8.

## 4 Discussion

For pure glucocorticoid action, the synthetic glucocorticoid dexamethasone (DEX) outperforms natural cortisol and corticosterone (Abd El-Hakam et al., 2022). Both males and females treated with DEX developed glucose and lipid intolerance, elevated levels of plasma insulin and triacylglycerol, increased amounts of glycogen and fat in the liver, and increased mass of islets in the pancreas (da Silva et al., 2023). Finding alternative remedies that are both safe and natural is, thus, a top priority. This work examined how SPAE protected rats’ livers from DEX-induced hyperlipidemia and injury.

In this study, administering dexamethasone (DEX) at a dose of 10 μg/kg/day for 4 weeks led to a noticeable suppression of food intake (FI) and a decline in body weight gain (BWG) in rats. This effect is likely associated with a dose-dependent rise in plasma leptin levels triggered by DEX, possibly contributing to reduced appetite and weight gain. Furthermore, it is proposed that leptin might be more involved in elevating energy expenditure rather than merely decreasing food consumption in DEX-induced anorexia (Won Jahng et al., 2008). This result was consistent with many reports indicating that DEX substantially reduced weight and increased fat mass (Li et al., 2017; Koorneef et al., 2022). Conversely, SPAE exhibited notable increases in feed intake (FI) and body weight gain (BWG) relative to the DEX group. The nutritional application of *Spirulina platensis* may enhance production performance, perhaps due to the high nutritional value of diets supplemented with *Spirulina* (Nassar et al., 2023; Cho et al., 2020; Aissaoui et al., 2017).

The positive effects on production performance may be due to the nutritional value of diets supplemented with *Spirulina platensis*, which is known for its high nutrient content (Kritsch et al., 2002; Wego et al., 2019). Moreover, Jiao et al. (2020) found that DEX considerably caused a swollen liver, medically known as hepatomegaly. They imply that lipid buildup could be associated with DEX-induced hepatomegaly. In contrast to the placebo group, SPAE reduces liver weight.

This data agrees with Hussein et al. (2020), who investigated the anti-obesity impact of SPAE in high-fat diet-induced obese rats. Also, Coué et al. (2019) exhibited that spirulina liquid extract alleviated western diet-induced non-alcoholic steatohepatitis, evidenced by a lower ratio of liver weight to body weight.

Dyslipidemia caused by DEX is associated with increased ROS and oxidative injury (Mohammed, 2021). Consistent with what Arab Dolatabadi and Mahboubi found, the present study found that injecting DEX considerably raised TC, TG, LDL, and VLDL while lowering HDL associated with the negative control group (Arab Dolatabadi and Mahboubi, 2015). One possible explanation for the observed shift from HDL to LDL following DEX treatment is increased hepatic lipogenesis due to increased fatty acid synthase and acetyl-CoA carboxylase expression (Cai et al., 2011). Rats administered SPAE improved lipid profiles in rats injected with DEX. Our data are supported by Hussein et al. (2020). The aqueous extract of SPAE dramatically reduced serum levels of total cholesterol, triacylglycerol, and LD. This lipid-lowering effect is likely linked to antioxidant-rich metabolites such as phycocyanin and phenolic acids, which may prevent fatty acid oxidation and lipolysis. Additionally, the polyunsaturated fatty acids in SPAE contribute to its anti-obesity properties. The extract’s essential fatty acids also help inhibit fat and cholesterol buildup. Similar outcomes were reported by Ouédraogo (2020). Researchers found that *spirulina* can lower lipid levels due to its high concentration of omega-3 and omega-6 fatty acids, beta-carotene, alpha-tocopherol, phycocyanin, phenols, and several minerals. There was a substantial elevation in liver enzyme activities in rats injected with DEX. These results are following Hasona and Morsi (2019). The results indicated that ALT and AST decreased extensively in the *Spirulina* group (Mazloomi et al., 2022). Daily supplementation with 1 g of *Spirulina platensis* for 12 weeks significantly improved lipid profiles in dyslipidaemic Cretan patients, notably reducing triglycerides, LDL-C, total cholesterol, and non-HDL-C levels. These results support the hypolipidaemic potential of *Spirulina* as a natural, well-tolerated dietary supplement and suggest its usefulness as an adjunct or alternative therapeutic strategy for managing dyslipidaemia, particularly in individuals at risk of cardiovascular disease (Mazokopakis et al., 2014).

GGt, T.BIL, and D.BIL levels were elevated in rats administered DEX. Our results agree with Alkot et al. (2022), confirming that DEX treatment caused a substantial increase in bilirubin levels. Potentially, oxidative stress causes alterations in cell membrane permeability, which alters the effective stress on the membrane, leading to elevated bilirubin levels (Itroutwar et al., 2020). In contrast, serum GGT, T.BIL, and D.BIL parameters significantly decreased in rats with SPAE. In harmony with these findings, Koite et al. (2022) tested the effects of a liquid Spirulina extract on metabolic abnormalities and oxidative stress in individuals suffering from metabolic syndrome.

DEX reduced total protein, albumin, and globulin (Mobeen et al., 2022; Ibrahim et al., 2020). The administration of DEX decreased serum total protein, albumin, and globulin levels (Savary et al., 2001). The decline in TP content can be useful in DEX regarding the severity of hepatocellular damage (Alavian et al., 2014). All groups administered with SPAE showed significant increases in serum proteins associated with the negative control group. The data are consistent with Youssef et al. (2023), who noticed that feeding 10.0% *Spirulina* substantially boosted the albumin serum level. El-Hashash (2021) reported that diet supplementation introduced to MSG–MSG-administered groups with Spirulina powder (0.5% and 1%) significantly increased total protein, albumin, and globulin. The normalized levels of serum proteins in SPAE contribute to phenolic chemicals in SP (Ramez et al., 2021). In aquatic models, *Spirulina platensis* enhanced haematological and serum biochemical parameters in rainbow trout (*Oncorhynchus mykiss*) (Yeganeh et al., 2015) and improved growth, immune response, and intestinal morphology in Nile tilapia (*Oreochromis niloticus*) (Al-Deriny et al., 2020). While occurring in different tissues and species, these benefits corroborate Spirulina’s consistent biological activity and role in metabolic modulation and immune resilience.

Lipid peroxidation is one of the basic mechanisms of tissue injury caused by free radicals (Mallikarjuna, 2018). In the present investigation, the injection of DEX led to the elevation of (MDA & NO) in the hepatic tissues of rats (Mallikarjuna, 2018). Also, our data are supported by Hasona and Morsi (2019); it was revealed that DEX treatment produced oxidative injury, as evidenced by the increased hepatic lipid peroxidation marker MDA, DEX-induced diminution of SOD and CAT in the hepatic tissue of rats. The reduction in both SOD and CAT activity may be associated with the suppression of enzyme protein production (Mallikarjuna, 2018; Alsadee and Agbashee, 2021). Abou-Seif et al. (2019) demonstrated that excessive levels of DEX significantly diminished overall antioxidant capacity and SOD activity, leading to oxidative stress through increased concentrations of hydrogen peroxide and MDA.

On the other hand, SPAE administration revealed significant decreases in (MDA & NO) and an important improvement in (CAT & SOD). Our findings, in line with previous findings by Al-Qahtani and Binobead (2019), SPAE treatment conferred significant, dose-dependent protection against liver oxidative stress caused by d-galactosamine (d-GalN) in rats, as demonstrated by elevated activity of the antioxidant enzymes SOD and CAT. The protective impact may be associated with the high concentration of antioxidant-rich metabolites in SPAE, such as C-phycocyanin, β-carotene, vital fatty acids, amino acids, vitamins, minerals, proteins, lipids, and carbs. These metabolites are recognized for their potent antioxidant and anti-inflammatory properties (Abdel-Daim et al., 2016). The biological activity of *Spirulina platensis* has been widely validated across multiple species, supporting its broad-spectrum protective effects. For instance, Zahran and Emam (2018) demonstrated that *Spirulina* ameliorated nicotine-induced renal oxidative stress and inflammation in rats, aligning with our hepatic findings regarding its anti-inflammatory and antioxidant properties. Similarly, Ardicli et al. (2022) reported modulation of stress-responsive HSP70 gene expression in rat brain tissue, suggesting systemic stress-mitigating effects that support our observed anti-apoptotic activity in the liver. The dietary supplementation with *Spirulina platensis* and its bioactive metabolites C-phycocyanin significantly improves lifespan and locomotor activity in a *Drosophila* model of Parkinson’s disease (DJ-1βΔ93). These beneficial effects are associated with modulation of oxidative stress markers and stress-responsive pathways, including HSP70 and JNK signaling. The findings highlight the potential of spirulina as a nutraceutical intervention for managing neurodegenerative disorders such as Parkinson’s disease through its antioxidant and cytoprotective properties (Kumar et al., 2017). These cross-species findings reinforce the mechanistic plausibility of our results and highlight *Spirulina platensis* as a robust nutraceutical candidate capable of modulating oxidative stress, apoptosis, and lipid metabolism in diverse biological systems.

Dexamethasone induces DNA damage and generates oxidative stress to exert an anti-cancer effect (Motafeghi et al., 2022). These results agree with (Motafeghi et al., 2022), who reported that dexamethasone has an anti-cancer effect by causing DNA damage and inducing oxidative injury. Also, the induction of DNA damage by Dexamethasone agrees with (Baumeister et al., 2009), who indicated that dexamethasone induced slight but considerable DNA fragmentation. Several research studies have reported the protective efficacy of *spirulina* against dexamethasone genotoxicity. Our results showed that *spirulina* has an effective role in reducing dexamethasone’s toxicity through reduced DNA damage. The high dose was more effective than the low dose, and both positively reduced DNA damage by reducing DNA tail and DNA moment.

Our results were confirmed by histopathological evaluation. In our results, dexamethasone’s induction of steatosis development agrees with (Hasona et al., 2017; Yin et al., 2017). They observed that an overdose of dexamethasone resulted in hyperglycemia, hyperlipidemia, the development of steatosis, and fatty liver. The present findings concur with (Abou-Seif and Hozayen, 2023); they asserted that dexamethasone precipitated pathological problems, including widespread fatty alteration in the hepatocytes within the hepatic parenchyma. The livers of rats administered DEX exhibited positive caspase-3 expression, which is recognized for its specific function in the ultimate triggering of apoptosis. These findings concur with those who indicated that dexamethasone induces oxidative stress and activates caspase 3, ultimately resulting in hepatotoxicity (Feng et al., 2017), who reported that treatment with Dex caused a remarkable increase in caspase-3 in osteoblasts.

SPAE’s protective effect is likely linked to its rich composition, including βPhycocyanin, selenium, β-carotene, and superoxide dismutase. Phycocyanin’s ability to prevent reactive metabolites and scavenge free radicals protects the liver (Ama Moor et al., 2017). Moreover, Al-Qahtani and Binobead (2019) Spirulina demonstrated a beneficial role in preserving liver function, offering protection against liver damage induced by harmful toxins when included in the diet.

This study explored the protective effects of *Spirulina platensis* aqueous extract against dexamethasone (DEX)-induced oxidative stress, apoptosis, lipid dysregulation, and DNA damage at the gene expression level. DEX significantly altered the expression of key regulatory genes, while Spirulina co-treatment modulated these alterations in a dose-dependent manner, suggesting its potential as a therapeutic nutraceutical. DEX treatment downregulated Nrf2 and SOD2, reflecting impaired antioxidant defense. *Nrf2* is a central transcription factor that activates cellular antioxidant genes via the Keap1–Nrf2–ARE signalling pathway (Kensler et al., 2007). The restoration and upregulation of Nrf2 and SOD2 by Spirulina, particularly at 800 mg/kg, suggest activation of antioxidant responses. These effects are likely attributed to Spirulina’s rich composition of phycocyanin, β-carotene, and polyphenols, known to counteract oxidative stress (Ibrahim et al., 2018). DEX significantly increased hepatic Keap1 expression, an established inhibitor of *Nrf2*, which restricts antioxidant gene transcription by sequestering *Nrf2* in the cytoplasm and promoting its degradation (Taguchi et al., 2011). SPAE treatment attenuated Keap1 expression, implying the release and nuclear translocation of *Nrf2*, thereby enhancing endogenous antioxidant defences. This mechanism is consistent with prior findings where *Spirulina*-derived metabolites promoted Nrf2 activation and reduced oxidative stress in hepatic injury models (Sibiya et al., 2022). Concurrently, SPAE upregulated AMPK expression, which was suppressed by DEX administration. AMPK is central to energy regulation, lipid oxidation, and inflammation control (Hardie, 2014). The observed AMPK upregulation aligns with studies showing that *Spirulina platensis* and its phycocyanin content can activate AMPK pathways, improving lipid metabolism and redox balance (Karimzadeh et al., 2025).

The therapeutic effects of *Spirulina* have been attributed to its rich content of bioactive metabolites, including phycocyanin, β-carotene, and phenolic acids, which exhibit strong antioxidant, anti-inflammatory, and lipid-lowering properties (Khan et al., 2005). These metabolites likely contributed to the modulation of Keap1 and AMPK pathways observed in our study.

Apoptosis markers were also significantly influenced by DEX. The upregulation of Bax and suppression of Bcl-2 in the DEX group indicate a shift toward pro-apoptotic signalling. The Bax/Bcl-2 ratio is a well-known indicator of apoptotic activity, and its normalization by Spirulina supports its anti-apoptotic capacity. These findings are consistent with previous research showing Spirulina’s inhibition of apoptosis in chemically induced hepatotoxicity (Amer et al., 2023). DEX-induced suppression of PPAR-α further suggests disrupted lipid metabolism. PPAR-α is critical in regulating fatty acid oxidation and maintaining lipid homeostasis (Desvergne et al., 2006). Spirulina’s ability to restore and enhance PPAR-α expression demonstrates its lipid-modulating potential, aligning with observed improvements in serum lipid profiles in related studies. Finally, p53 was significantly upregulated following DEX exposure, indicating increased cellular stress and DNA damage. As a tumour suppressor and regulator of cell cycle arrest and apoptosis, p53 activation follows oxidative or genotoxic insults (Vousden and Lane, 2007). Spirulina co-treatment significantly reduced p53 expression, suggesting DNA damage and cellular stress mitigation. These findings highlight the protective, multi-targeted effect of *Spirulina platensis* against glucocorticoid-induced molecular damage. Modifying gene expression across antioxidant, apoptotic, metabolic, and genomic stress pathways reinforces its promise as a natural therapeutic agent.

While the 28-day treatment period and use of only male rats were consistent with standard protocols to reduce variability in early-phase toxicological studies, these choices limit the extrapolation of our findings to chronic exposure scenarios and across sexes. Future studies should adopt longer treatment durations and include both male and female animals to assess the sustained effects and potential sex-specific responses to *Spirulina platensis*. Additionally, although we observed significant modulation of Nrf2, PPAR-α, and p53 gene expression, our study does not establish direct causality. Mechanistic confirmation using pathway-specific inhibitors or genetic models will be essential in future work to delineate the specific molecular targets underlying SPAE’s protective actions. Although the *Spirulina platensis* powder was obtained from a reputable supplier and analyzed by HPLC, the extract lacked standardization to a specific bioactive marker. Future studies should standardize key metabolites like phycocyanin or total phenolics to enhance reproducibility and pharmacological consistency. Although alterations in Bax, Bcl-2, and caspase-3 expression strongly indicate activation of the intrinsic apoptotic pathway, mitochondrial integrity or membrane potential (ΔΨm) was not directly assessed in this study. Future investigations are warranted to confirm these findings using mitochondrial-specific assays to elucidate further the mechanisms by which *Spirulina platensis* mitigates DEX-induced hepatotoxicity.

## 5 Conclusion

The antioxidant effects of *Spirulina platensis* aqueous extract (SPAE) contributed to regulating biochemical parameters, including liver enzymes and lipid profiles, in male rats subjected to dexamethasone-induced oxidative stress. Additionally, SPAE modulated the expression of key genes involved in oxidative stress response (*Nrf2*, *SOD2*), apoptosis (*Bax*, *Bcl-2*), lipid metabolism (*PPAR-α*), and DNA damage (*p53*), further supporting its protective role at the molecular level. These findings highlight the potential of SPAE as a functional metabolite in food and beverage formulations aimed at enhancing antioxidant defences and mitigating glucocorticoid-induced metabolic disturbances. Further research is warranted to validate its efficacy and safety for human consumption.

## Data Availability

The original contributions presented in the study are included in the article/Supplementary Material, further inquiries can be directed to the corresponding authors.
