# Supplementary material for: Modulatory role of Spirulina platensis in oxidative stress, apoptosis, and gene expression in a rat model of dexamethasone-induced hepatotoxicity
Source: Front Pharmacol. 2025 Aug 18;16:1610793. doi: 10.3389/fphar.2025.1610793 (PMC12399871; doi:10.3389/fphar.2025.1610793)
Supplement: Supplementary file 1 [file DataSheet2.pdf]

Table S1: Rat-Specific qPCR Primer Details

Gene	Accession Number	Forward Primer (5'→3')	Reverse Primer (5'→3')	Amplicon Size (bp)	Efficiency (%)
Nrf2	NM_001399173.1	GCACATCCAGACAGACACCA	CTCTCAACGTGGCTGGGAAT	189	95.8%
SOD2	NM_017051.2	GCTGGCCAAGGGAGATGTTA	TGTGATTGATATGGCCCCCG	80	94.5%
Bax	NM_017059.2	CTCAAGGCCCTGTGCACTAA	GGAAAGGAGGCCATCCCAG	126	96.2%
Bcl-2	NM_016993.1	CTGGTGGACAACATCGCTCT	GCATGCTGGGGCCATATAGT	115	93.7%
PPAR-α	NM_013196.2	GGCTCTGAACATTGGCGTTC	CAAGGGGACAACCAGAGGAC	96	95.1%
p53	NM_030989.3	GGTTCGTGTTTGTGCCTGTC	TGCTCTCTTTGCACTCCCTG	109	94.8%
β-Actin	NM_031144.3	CTGTGTGGATTGGTGGCTCT	AGCTCAGTAACAGTCCGCC	134	97.3%
Keap1	NM_057152.2	CCCTGAACTCCTACCACCCA	TACCCCGAAACAGACAGACAAC	471	92.3%
AMPK	NM_023991	GGTAGGGGAGACCACCTCAT	CTGCATCCTTCCTGCCTTCA	127	95.5%

Table S2: Phenolic profile of *Spirulina platensis* (µg/g sample)

**Compound**	**Algae**
Gallic acid	1.38
Protocatechuic acid	1.50
p-hydroxybenzoic acid	3.76
Gentisic acid	0.53
Cateachin	44.53
Chlorogenic acid	9.51
Caffeic acid	1.08
Syringic acid	ND
Vanillic acid	0.35
Ferulic acid	0.46
Sinapic acid	ND
p-coumaric acid	0.36
Rutin	ND
Rosmarinic acid	0.94
Apigenin-7-glucoside	4.14
Cinnamic acid	0.80
Qurecetin	1.01
Apigenin	0.64
Kaempferol	0.66
Chrysin	2.15

ND = Not detected

Method:

HPLC analysis was carried out according to **Kim *et al.* (2006)** using Agilent Technologies 1100 series liquid chromatograph equipped with an auto sampler and a diode-array detector. The analytical column was a Eclipse XDB-C18 (150 X 4.6 µm; 5 µm) with a C18 guard column (Phenomenex, Torrance, CA). The mobile phase consisted of acetonitrile (solvent A) and 2% acetic acid in water (v/v) (solvent B). The flow rate was kept at 0.8 ml/min for a total run time of 60 min and the gradient programme was as follows: 100% B to 85% B in 30 min, 85% B to 50% B in 20 min, 50% B to 0% B in 5 min and 0% B to 100% B in 5 min. The injection volume was 20 µl and peaks were monitored simultaneously at 280, 320 and 360 nm. All samples were filtered through a 0.45 µm Acrodisc syringe filter (Gelman Laboratory, MI) before injection. Peaks were identified by congruent retention times and UV spectra and compared with those of the standards.

Kim, K. H.; Tsao, R.; Yang, R. and Cui, S. W. (2006). Phenolic acid profiles and antioxidant activities of wheat bran extracts and the effect of hydrolysis conditions. Food Chemistry, 95: 466-473.
