# Supplementary material for: Modulatory role of Spirulina platensis in oxidative stress, apoptosis, and gene expression in a rat model of dexamethasone-induced hepatotoxicity
Source: Front Pharmacol. 2025 Aug 18;16:1610793. doi: 10.3389/fphar.2025.1610793 (PMC12399871; doi:10.3389/fphar.2025.1610793)
Supplement: Supplementary file 2 [file DataSheet1.pdf]

1g in 2ml

===

Injection Date : 8/24/2021 5:46:50 PM
Sample Name : Algea Location : Vial 34
Acq. Operator : A
Acq. Instrument : Instrument 1 Inj Volume : 20 µl
Acq. Method : C:\HPCHEM\1\METHODS\PHENOLS2.M
Last changed : 8/24/2021 5:45:30 PM by A
(modified after loading)
Analysis Method : C:\HPCHEM\1\METHODS\PHENOLS2.M
Last changed : 8/24/2021 6:55:49 PM by A
(modified after loading)

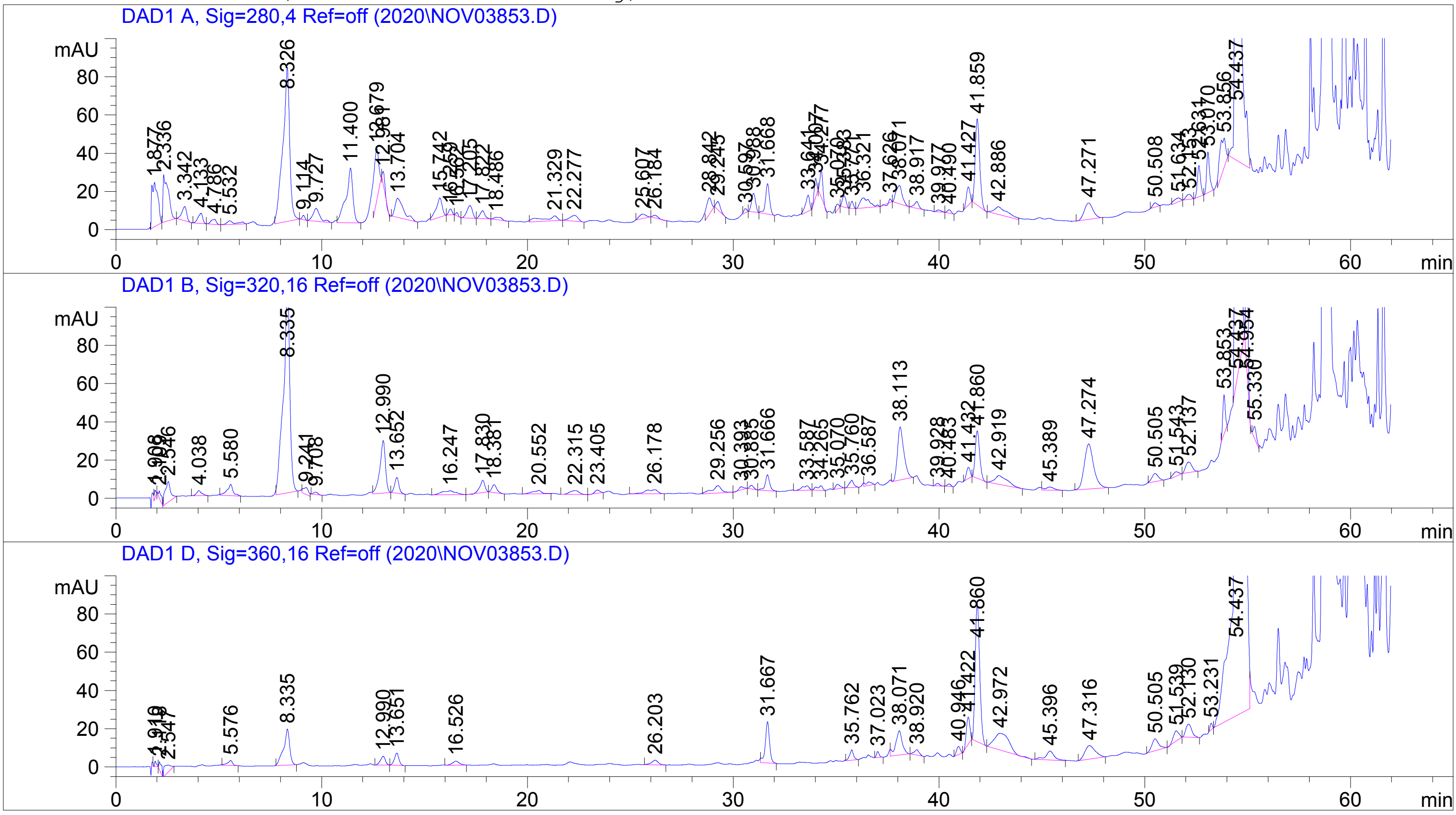

===

Area Percent Report

===

Sorted By : Signal
Multiplier : 1.0000
Dilution : 1.0000
Use Multiplier & Dilution Factor with ISTDs

Signal 1: DAD1 A, Sig=280,4 Ref=off

Peak #	RetTime [min]	Type	Width [min]	Area [mAU*s]	Height [mAU]	Area %
1	1.877	PB	0.2530	462.29971	23.14960	3.4566
2	2.336	VB	0.2390	444.17737	24.10107	3.3211
3	3.342	VBA	0.2570	122.73504	7.41836	0.9177
4	4.133	PB	0.2550	83.02629	5.29455	0.6208
5	4.786	VB	0.2361	44.57248	2.59886	0.3333
6	5.532	VBA	0.3826	54.80805	1.95800	0.4098
7	8.326	BBA	0.3270	1860.71216	80.10269	13.9124
8	9.114	VB	0.1480	21.04816	2.27376	0.1574
9	9.727	VBA	0.3511	156.02841	6.71383	1.1666
10	11.400	BBA	0.3224	670.17523	28.49673	5.0108
11	12.679	PB	0.2336	409.75360	26.91150	3.0637
12	12.981	VBA	0.1527	52.06772	5.80203	0.3893
13	13.704	PBA	0.4637	286.04425	9.93991	2.1387
14	15.742	PB	0.2972	206.40627	9.98289	1.5433
15	16.259	VB	0.1740	34.60573	3.10519	0.2587

Peak #	RetTime [min]	Type	Width [min]	Area [mAU*s]	Height [mAU]	Area %
16	16.562	VB	0.1401	14.17124	1.65179	0.1060
17	17.205	VB	0.2907	121.02877	6.57749	0.9049
18	17.822	VBA	0.2480	66.40596	4.12002	0.4965
19	18.486	VBA	0.3531	32.32421	1.23618	0.2417
20	21.329	PB	0.6670	125.53265	2.49493	0.9386
21	22.277	VBA	0.4549	85.90823	3.06575	0.6423
22	25.607	BB	0.3743	51.04670	2.27082	0.3817
23	26.184	VBA	0.2582	22.94181	1.37808	0.1715
24	28.842	PB	0.2685	144.15782	8.55737	1.0779
25	29.245	VBA	0.2296	59.57957	4.19781	0.4455
26	30.597	BB	0.1606	22.22394	2.22425	0.1662
27	30.988	VB	0.2126	130.40584	9.71425	0.9750
28	31.668	VBA	0.2009	214.00632	15.91215	1.6001
29	33.641	PB	0.2055	116.25134	8.39931	0.8692
30	34.007	VB	0.1390	82.33725	9.70790	0.6156
31	34.277	VBA	0.1479	129.50380	14.00524	0.9683
32	35.070	PB	0.1736	31.07743	3.08379	0.2324
33	35.383	VB	0.2120	73.52054	5.79680	0.5497
34	35.771	VB	0.1383	33.04831	3.77014	0.2471
35	36.321	VB	0.4392	167.59528	5.32456	1.2531
36	37.626	VB	0.0874	8.23097	1.86648	0.0615
37	38.071	VB	0.2908	196.25836	9.58682	1.4674
38	38.917	VBA	0.2169	51.89295	3.50207	0.3880
39	39.977	PB	0.2450	15.84823	1.02167	0.1185
40	40.490	VBA	0.2015	21.83924	1.75122	0.1633
41	41.427	PB	0.1986	116.87154	9.56461	0.8738
42	41.859	VBA	0.2361	680.15161	45.04903	5.0854
43	42.886	BBA	0.6154	185.57242	4.04370	1.3875
44	47.271	PBA	0.4641	253.33553	8.58956	1.8942
45	50.508	PBA	0.2504	33.22369	2.17277	0.2484
46	51.634	PB	0.4067	48.53986	2.03216	0.3629
47	52.153	VB	0.3007	44.88810	2.55855	0.3356
48	52.631	VB	0.1627	177.70326	16.36836	1.3287
49	53.070	VBA	0.1560	218.88486	21.27850	1.6366
50	53.856	PB	0.2979	352.83325	15.48261	2.6381
51	54.437	VBA	0.2359	4336.90674	258.25052	32.4267

Totals : 1.33745e4 754.45624

Results obtained with enhanced integrator!

Signal 2: DAD1 B, Sig=320,16 Ref=off

Peak #	RetTime [min]	Type	Width [min]	Area [mAU*s]	Height [mAU]	Area %
1	1.908	BB	0.0834	21.68546	3.87111	0.1711
2	2.109	VB	0.1481	41.56086	3.67019	0.3280
3	2.546	VBA	0.2554	200.44417	10.85534	1.5817
4	4.038	BBA	0.2399	46.41507	2.70867	0.3663
5	5.580	BBA	0.2796	122.79434	5.97966	0.9690
6	8.335	BBA	0.2930	2371.29199	112.90404	18.7121
7	9.241	BBA	0.2490	64.68264	3.68073	0.5104
8	9.708	BBA	0.2261	16.44506	1.18367	0.1298
9	12.990	BB	0.2775	521.47656	27.47171	4.1150
10	13.652	VBA	0.2213	120.15807	8.28160	0.9482
11	16.247	PB	0.6109	98.21722	2.06500	0.7750
12	17.830	VB	0.2490	105.93730	6.40471	0.8360
13	18.381	VBA	0.2584	68.14623	4.09067	0.5377
14	20.552	PBA	0.4275	52.54269	1.68656	0.4146
15	22.315	PB	0.5030	62.33665	2.02574	0.4919
16	23.405	VBA	0.2564	26.13147	1.65310	0.2062
17	26.178	BBA	0.5898	98.46289	2.21840	0.7770
18	29.256	PB	0.4555	125.24411	3.89117	0.9883
19	30.393	VB	0.2349	25.86151	1.68626	0.2041
20	30.885	VB	0.2154	25.07471	1.83517	0.1979
21	31.666	VBA	0.2238	123.60252	8.20255	0.9754
22	33.587	BBA	0.3382	56.22997	2.26069	0.4437
23	34.265	VBA	0.2681	46.67851	2.38717	0.3683
24	35.070	PB	0.2416	37.88953	2.43430	0.2990
25	35.760	VBA	0.2141	58.39084	3.82415	0.4608
26	36.587	BBA	0.2686	36.90422	1.85071	0.2912

Peak #	RetTime [min]	Type	Width [min]	Area [mAU*s]	Height [mAU]	Area %
27	38.113	BBA	0.3247	599.22278	27.66683	4.7285
28	39.928	PBA	0.2014	18.18213	1.41979	0.1435
29	40.483	VBA	0.1943	14.76717	1.24615	0.1165
30	41.432	PB	0.2043	72.60168	5.87062	0.5729
31	41.860	VBA	0.2334	370.44626	24.92640	2.9232
32	42.919	VBA	0.5873	201.71336	4.63857	1.5917
33	45.389	BBA	0.4324	46.52016	1.57591	0.3671
34	47.274	PBA	0.4909	747.45856	23.51315	5.8983
35	50.505	BBA	0.3426	92.09057	4.21973	0.7267
36	51.543	PB	0.3374	46.56047	2.25033	0.3674
37	52.137	VBA	0.3460	118.02596	5.50801	0.9314
38	53.853	BBA	0.1353	177.13390	20.82178	1.3978
39	54.437	PB	0.1946	4951.44580	373.54510	39.0723
40	54.954	VBA	0.1082	573.50317	81.41769	4.5256
41	55.330	BBA	0.1677	68.24622	6.13806	0.5385

Totals : 1.26725e4 813.88120

Results obtained with enhanced integrator!

Signal 3: DAD1 D, Sig=360,16 Ref=off

Peak #	RetTime [min]	Type	Width [min]	Area [mAU*s]	Height [mAU]	Area %
1	1.910	BB	0.0978	19.17734	2.80472	0.0884
2	2.118	VB	0.1417	44.18602	4.10090	0.2036
3	2.547	VBA	0.3317	96.23656	3.79680	0.4434
4	5.576	BBA	0.2458	45.08681	2.60713	0.2077
5	8.335	BBA	0.2897	389.33813	18.79346	1.7937
6	12.990	PB	0.2568	77.85657	4.61577	0.3587
7	13.651	VBA	0.2280	96.87964	6.42147	0.4463
8	16.526	BBA	0.3465	49.55219	2.10577	0.2283
9	26.203	BBA	0.3302	53.56299	2.41985	0.2468
10	31.667	BBA	0.2169	311.47733	21.51706	1.4350
11	35.762	PBA	0.1893	69.91152	5.31981	0.3221
12	37.023	PBA	0.1374	28.69449	3.17662	0.1322
13	38.071	BB	0.3294	287.73782	12.64702	1.3256
14	38.920	VBA	0.2416	46.59953	2.80795	0.2147
15	40.946	PB	0.1949	52.51885	4.28731	0.2420
16	41.422	VB	0.1937	168.82510	14.30218	0.7778
17	41.860	VB	0.2381	1108.14063	72.58153	5.1054
18	42.972	VBA	0.8067	450.86639	8.78615	2.0772
19	45.396	BBA	0.4330	138.42685	4.68075	0.6378
20	47.316	PBA	0.5389	246.80067	7.00690	1.1370
21	50.505	PB	0.3517	144.40251	6.20009	0.6653
22	51.539	VB	0.3186	99.40729	5.21010	0.4580
23	52.130	VBA	0.3270	141.20786	6.89306	0.6506
24	53.231	PB	0.1299	27.34067	3.54731	0.1260
25	54.437	VBA	0.2485	1.75112e4	980.07764	80.6766

Totals : 2.17054e4 1206.70735

Results obtained with enhanced integrator!

===

*** End of Report ***
